# Neural correlates of breath work, mental imagery of yoga postures, and meditation in yoga practitioners: a functional near-infrared spectroscopy study

**DOI:** 10.3389/fnins.2024.1322071

**Published:** 2024-03-21

**Authors:** Xiawen Li, Yu Zhou, Chenping Zhang, Hongbiao Wang, Xiaochun Wang

**Affiliations:** ^1^Shanghai University of Medicine and Health Sciences, Shanghai, China; ^2^Shanghai University of Sport, Shanghai, China

**Keywords:** yoga, breath, meditation, posture, fNIRS

## Abstract

**Objective:**

Previous research has shown numerous health benefits of yoga, a multicomponent physical and mental activity. The three important aspects of both traditional and modern yoga are breath work, postures, and meditation. However, the neural mechanisms associated with these three aspects of yoga remain largely unknown. The present study investigated the neural underpinnings associated with each of these three yoga components in long- and short-term yoga practitioners to clarify the neural advantages of yoga experience, aiming to provide a more comprehensive understanding of yoga’s health-promoting effects.

**Methods:**

Participants were 40 Chinese women, 20 with a long-term yoga practice and 20 with a short-term yoga practice. Functional near-infrared spectroscopy was conducted while participants performed abdominal breathing, mental imagery of yoga postures, and mindfulness meditation. The oxygenated hemoglobin concentrations activated in the brain during these three tasks were used to assess the neural responses to the different aspects of yoga practice. The self-reported mastery of each yoga posture was used to assess the advantages of practicing yoga postures.

**Results:**

Blood oxygen levels in the dorsolateral prefrontal cortex during breath work were significantly higher in long-term yoga practitioners than in short-term yoga practitioners. In the mental imagery of yoga postures task, self-reported data showed that long-term yoga practitioners had better mastery than short-term practitioners. Long-term yoga practitioners demonstrated lower activation in the ventrolateral prefrontal cortex, with lower blood oxygen levels associated with performing this task, than short-term yoga practitioners. In the mindfulness meditation task, blood oxygen levels in the orbitofrontal cortex and the ventrolateral prefrontal cortex were significantly higher in long-term yoga practitioners than in short-term yoga practitioners.

**Conclusion:**

The three core yoga components, namely, yogic breathing, postures, and meditation, showed differences and similarities in the activation levels of the prefrontal cortex. Long-term practice of each component led to the neural benefits of efficient activation in the prefrontal cortex, especially in the dorsolateral prefrontal cortex, orbitofrontal cortex, and ventrolateral prefrontal cortex.

## Introduction

Yoga, a multicomponent physical and mental activity, is increasingly favored by the public as a healthy practice. Practicing yoga can not only improve physical fitness but also help the practitioner achieve a state of mental balance, a sense of inner peace, and more harmonious interactions with the external environment (Brinsley et al., 2022; Joshanloo, 2022; Cagas et al., 2023). There are many varieties of yoga styles practiced worldwide. Although each yoga style has its own characteristics, evidence has shown that breath work, postures, and meditation are the three core components of almost all yoga classes (Mandlik et al., 2023). Currently, there is substantial evidence indicating that the behavioral effects of these three yoga components are not exactly the same, and each possesses its distinct set of benefits (Prado et al., 2014; Domingues, 2018; Vonderlin et al., 2020; Das et al., 2021; Maleki et al., 2022). However, the neural underpinnings of the three yoga components remain largely unknown. Thus, the present study aimed to examine the specific neural correlates associated with each of the three components within a single study.

Although little is known about the underlying neural characteristics of the yoga components, many researchers have compared the explicit effects of the separate yoga components. Semich (2012) explored how performing various postures only or a having a combined yoga practice of breath work, postures, and meditation affected perceived stress levels among the participants. They found that multi-component yoga was more effective than performing yoga postures alone to lower perceived stress levels (Semich, 2012). Wheeler et al. (2019) examined the individual effects of postures, breath work, and meditation on stress responses and found that these three yoga aspects reduced state anxiety with no difference between the three components. Studies associated with breath work have found that breath training can enhance lung function and reduce anxiety (Kupershmidt and Barnable, 2019; Maleki et al., 2022). Yoga posture training has been shown to correct poor body posture (Jorrakate et al., 2015), increase body balance (Prado et al., 2014), and decrease anxiety (Domingues, 2018). Studies associated with meditation show that meditation effectively relieves emotional problems and improves well-being (Vonderlin et al., 2020). Thus, these study results indicated that all three components not only have similar benefits, such as reducing emotional distress, but also have their own unique advantages.

Changes in explicit performance are often a result of corresponding changes in neural activity. Revealing how changes at the neural level are elicited by practicing specific yoga aspects can facilitate the development of yoga interventions in both healthy and clinical populations. In recent years, a few studies have used functional near-infrared spectroscopy (fNIRS) technology to explore the neural correlates of each of the three yoga components separately. In research on yogic breathing, Bhargav et al. (2014) observed significant changes in prefrontal cortex (PFC) activity after high frequency yogic breathing in healthy people. Singh et al. (2016) measured the effect of uninostril yogic breathing on PFC hemodynamics. They observed that right nostril yogic breathing increased activity in the left PFC more than left nostril yogic breathing (Singh et al., 2016). However, the neural underpinnings, as assessed by fNIRS, associated with yogic abdominal breathing remain largely unknow. The present study aimed to address this gap. Yogic breathing consists of a variety of styles in which slow and deep abdominal breathing is a basic and core technique. Yildiz et al. (2022) examined the impact of four yogic breath styles on brain health using a 3 T magnetic resonance imaging system. They found that the assessed meditator of brain health changes greatest during abdominal breathing (Yildiz et al., 2022). Burt et al. (2023) used fNIRS to find that activity in the PFC increases with increased breathing effort. Since abdominal breathing requires more effort in abdominal muscular recruitment than natural breathing (Bahensky et al., 2021), we hypothesized that the PFC hemodynamics during abdominal breathing would increase.

Other studies have focused on posture-based yoga. Chen et al. (2021) used fNIRS to find that yoga posture training, such as practicing the tree pose, activates the supplementary motor area, improving balance on one leg, which can be used as an exercise therapy for people with impaired balance (Chen et al., 2021). Dybvik and Steinert (2021) used fNIRS to investigate brain activity during yoga posture practice. They found differences in prefrontal activation when comparing simple postures to complex postures, differences that represented different cognitive loads (Dybvik and Steinert, 2021). The postures used in these studies are relatively simple; a novice with no practice experience could also complete these postures with guidance. Long-term yoga practitioners can perform many difficult postures with increased posture practice over time that short-term practitioners cannot. Since long-term exercise facilitates neuroplasticity of certain brain functions (Hötting and Röder, 2013), how different brain activities are evoked by these more difficult postures between long-term and short-term yoga practitioners may provide better understanding of the neural characteristics associated with yoga posture practice. Thus, the present study used mental imagery of yoga postures to assess whether neural activity differed between difficult vs. simple yoga postures in long-and short-term yoga practitioners.

Numerous clinical studies using fNIRS have shown that mindfulness meditation interventions can relieve emotion problems (Gundel et al., 2018) and improve attention and cognitive control performances (Gao and Zhang, 2023). Choo et al. (2019) highlighted the role of PFC activity during mindfulness meditation. Gao and Zhang (2023) observed a stronger activation of the dorsolateral PFC (DLPDC) during mindfulness meditation. But few studies have explored the neural responses to yogic mindfulness meditation. Jiang et al. (2021) found that brain activity in the PFC increased during an inhibitory control task after a yogic mindfulness meditation intervention. At present, there is limited research examining how PFC hemodynamics change during yogic mindfulness meditation. The effect of yogic mindfulness meditation practice experience on PFC activity also remains unclear.

Thus, the present study compared neural changes during abdominal breathing, mental imagery of postures, and mindfulness meditation between long-and short-term yoga practitioners to further explore the separate neural responses to each yoga component. The study analyzed concentration changes in oxyhemoglobin caused by neural activation in the DLPFC, ventrolateral PFC (VLPFC), and orbitofrontal cortex. The DLPFC is important for regulating attention and is closely related to cognitive function (Mooneyham et al., 2016). The orbitofrontal cortex (OFC) has a wide connection with the emotional center and is closely related to emotion regulation (Kral, 2020). The VLPFC participates in self-related processing (Joshanloo, 2022) and motor activity (Selleck et al., 2018). As previously stated, the results of the aforementioned studies indicate that the three yoga components are associated with decreases in negative emotion and increases in attention focus and cognitive and motor control performances. Each of these functions is controlled largely by the various subdivisions of the PFC. Thus, we hypothesized that experienced yoga practitioners would show similar but also unique neural activities in the various subdivisions of the PFC during breath work, mental imagery of yoga postures, and mindfulness meditation. We also hypothesized that practice experience in the three yoga components would enhance neural activity in the corresponding PFC area.

## Materials and methods

### Participants

A total of 40 women were recruited from yoga studios in China: 20 long-term yoga practitioners and 20 short-term yoga practitioners. All participants were native Chinese women between 18 and 40 years of age. Long-term yoga practitioners practiced yoga more than 3 times a week for a mean (SD) of 6.05 (1.39) years, whereas short-term yoga practitioners practiced yoga more than 3 times a week for a mean (SD) of 0.91 (0.38) years. Table 1 summarizes the demographic characteristics of the participants. This study was approved by the Shanghai University of Sport Ethics Committee and was performed in accordance with the ethical standards laid down in the 1964 Declaration of Helsinki and its later amendments. All participants provided written informed consent prior to the study.

**Table 1:** Demographic and yoga training characteristics of participants.

Characteristic (years)	Long-term practitioners		Short-term practitioners		*t*-test scores
	Mean	SD	Mean	SD	
Age	31.95	7.13	28.50	6.99	0.13
Educational level	14.55	1.87	15.30	1.78	0.20
Yoga training	6.05	1.39	0.91	0.38	<0.01

### Stimuli

The present study selected 30 yoga posture images. Of them, 15 were simple postures and 15 were difficult postures. Each difficult posture was an advanced version of the corresponding simple posture, and there were some similarities in the activation of the muscles between the simple and difficult postures. All images were consistent in size and luminance. Table 2 gives the names of the postures that were used.

**Table 2:** Postures used for performing mental imagery of yoga postures.

Simple postures	Difficult postures
Mountain pose with arms up	Handstand pose
Downward facing dog pose	Headstand pose
Plant pose	Crane pose
Locust pose	Full locust
Cat pose	Scorpion pose
Tree pose	Standing sun dial pose
Cobra pose	Snake king pose
Wide legged forward bend	Tortoise pose
Imaginary chair pose	Upsidedown chair
Forearm plank pose	Forearm balance
Pigeon pose	Hanuman pose
Triangle pose	Extend hand to big toe pose
dancer pose	Lord of the dance pose
bridge pose	Wheel pose
Skyscraper pose	Tiptoe pose

### Procedure

The experiment consisted of three tasks: abdominal breathing (task 1), mental imagery of yoga posture (task 2), and mindfulness meditation (task 3). After the participants had been introduced to the experiment, the fNIRS instrumentation (NIRSport2, NIRx, Germany) was placed on them. Participants performed these three tasks while fNIRS data were recorded. The three tasks were compiled and run using E-Prime 3.0 software (Psychology software tools, INC, America). The three tasks were presented in a random order.

**Figure 1:**
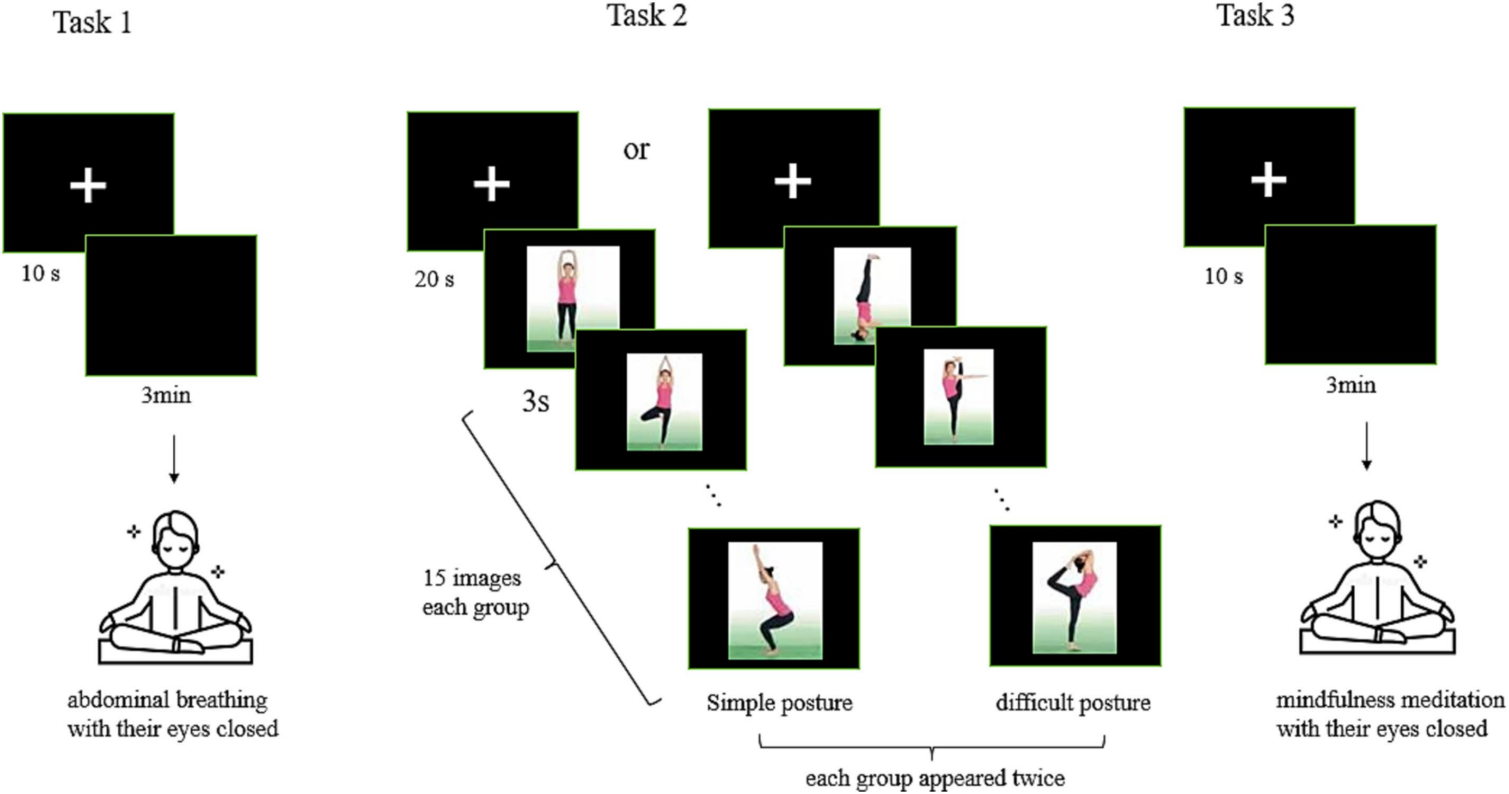
Procedures for the three yoga component tasks.

Task 1 began with a 10-s fixation screen appearing on a monitor, followed by a black blank screen for 3 min. During those 3 min, participants were instructed to practice abdominal breathing with their eyes closed, keeping the breath slow and deep and coming from the abdomen. They were further instructed that as they reached the end of each inhalation to begin exhaling without holding their breath (see Figure 1).

Task 2 began with a 20-s fixation screen, followed by the presentation of images of 15 different postures, each of which was presented for 3 s. Participants were instructed to view each image and imagine the activation of their body muscles as though they were performing each posture themselves. The experiment consisted of two groups of posture images: one group of 15 simple postures and one group of 15 difficult postures (Table 2). The order in which these two groups of images was presented was random, and each image in each group appeared twice. Participants were then asked to view these images again and to self-rate their mastery of each posture using the following scale: 1 represented not at all; 2, occasionally; 3, relatively easy; and 4, very easy. We did not conduct fNIRS while the participants self-reported their mastery of each posture (see Figure 1).

Task 3 began with a 10-s fixation screen, followed by a black blank screen for 3 min. During those 3 min, participants were asked to practice open monitoring mindfulness meditation. The participants were instructed to close their eyes while attempting to focus attention and stay aware of the present moment, thoughts, feelings without any judgement, that is, maintaining an open and receptive attitude to the moment (Kabat-Zinn, 2021) (see Figure 1).

### Data acquisition and analyses

**Figure 2:**
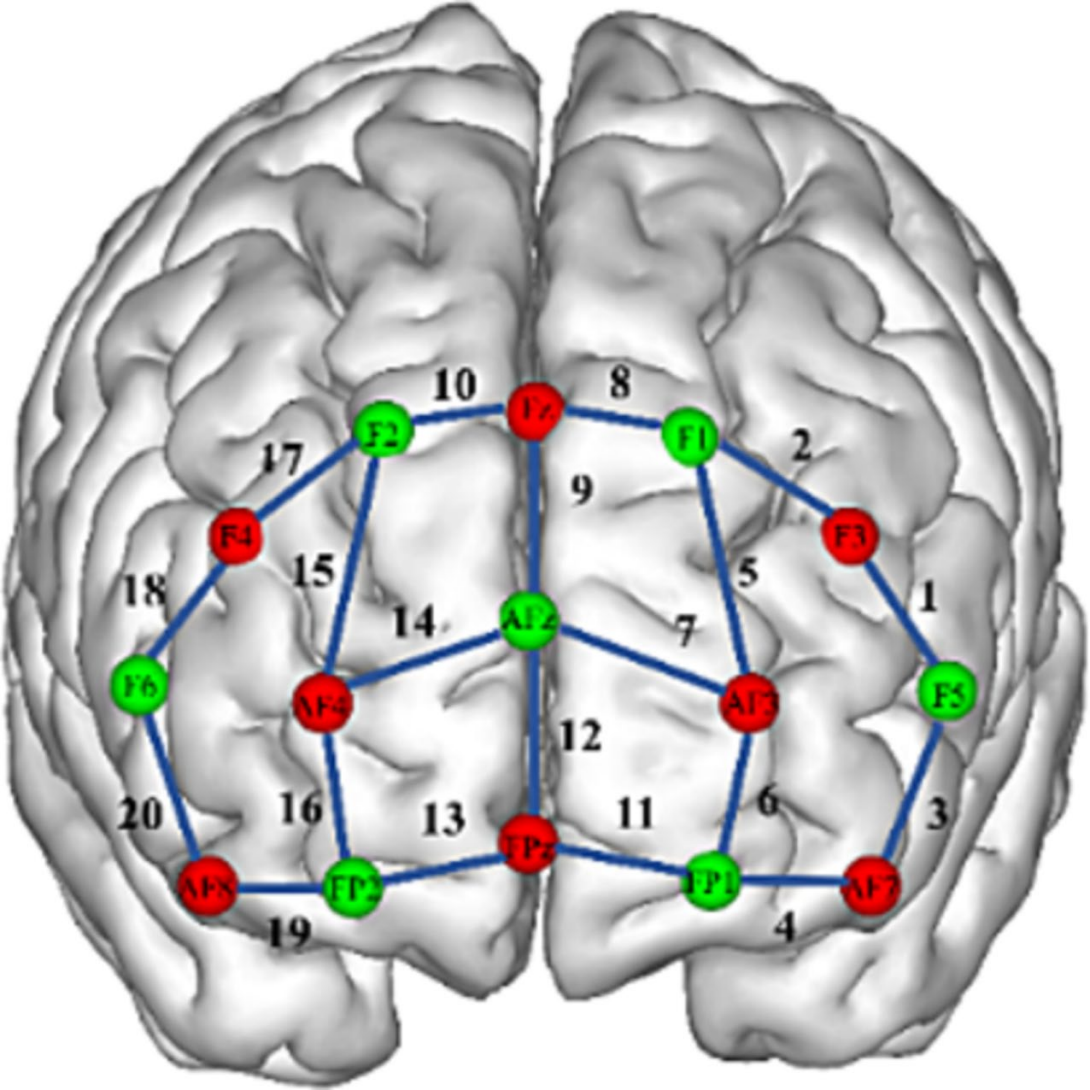
Each channel corresponds to an area within the prefrontal cortex.

The fNIRS data were collected with the NIRSport2 system (NIRx, Germany). We acquired 760 and 850 nm dual-wavelength near-infrared light to measure the relative concentration changes of oxyhemoglobin and deoxyhemoglobin (Yamashita et al., 1996) based on the modified Beer–Lambert law (Cope et al., 1988) with a sampling frequency of 10 Hz. For the fNIRS experiment, eight sources and seven detectors (yielding 20 channels) were placed over the PFC region. The distance between the source and the detector was 3 cm. Sensors were located by aligning the bottom row of electrodes with the International 10–20 AF7-Fp1-Fpz-Fp2-AF8 line (Jurcak et al., 2007). Researchers have identified a correspondence between the location of fNIRS channels and specific brain regions (Okamoto et al., 2004; Tsuzuki et al., 2007). In the present study, the ventrolateral prefrontal cortex (VLPFC) was represented by channels 1, 3, 18, and 20; the DLPFC, by channels 2, 5, 8, 9, 10, 15, and 17; the OFC, by channels 4, 11, 13, and 19; and the frontopolar prefrontal cortex (FPA), by channels 6, 7, 12, 14, and 16 (see Figure 2).

The fNIRS data were evaluated with Homer2 software (MGH-Martinos Center for Biomedical Imaging, Boston, MA, United States) using MATLAB (MathWorks, Natick, MA, United States). Motion artifacts were detected as signal changes more by than10% of the standard deviation of the signal within 0.5 s and were removed by wavelet filtering (Molavi and Dumont, 2012). Baseline drift was removed using a high-pass filter with a cutoff frequency of 0.01 Hz, and a low-pass filter with a frequency of 0.1 Hz was used to reduce the impact of the heartbeat, respiration, blood pressure, and skin blood flow signals. The changes in the oxygenated hemoglobin (HbO) concentration were calculated using the modified Beer–Lambert law. The average response of each participant in the three tasks at 20 channels was obtained by using the block average (Strangman et al., 2002).

### Statistical analysis

Statistical analyses were performed in SPSS, version 22.0 (IBM Inc.). The independent samples t-test was used to determine the difference between two groups in the HbO concentration at 20 channels in the PFC during breath work and meditation. The self-reported mastery of each yoga posture and the HbO concentration activated in the PFC while processing that yoga posture were analyzed by two-way repeated-measures analyses of variance (ANOVAs), with main effects of group (long-term vs. short term yoga practitioners) and posture difficulty level (simple vs. difficult). The Benjamini-Hochberg false discovery rate procedure was used for the fNIRS data (Benjamini and Hochberg, 1995). Values are presented as means ± SDs. Differences with 2-sided *p*-values <0.05 were considered statistically significant.

## Results

### Task 1: breath work

The results of independent samples t-tests showed that the HbO concentration in the DLPFC (channel 9) of long-term yoga practitioners was significantly higher than that of short-term yoga practitioners (*p* = 0.014) during abdominal breathing. The increased activation in the DLPFC during breath work in long-term yoga practitioners may suggest a benefit of yogic breathing on DLPFC function (see Table 3).

**Table 3:** Mean changes in HbO concentration assessed in four prefrontal cortical areas through 20 fNIRS channels during abdominal breathing between long-and short-term yoga practitioners.

		Long-term practitioners		Short-term practitioners					
Area	Channel	Mean/μm	SD	Mean/μm	SD	*t*	*p*-value	Corrected-p	Cohen’s d
OFC									
	4	0.0063	0.0204	−0.0020	0.0194	1.308	0.199	0.796	0.417
	11	0.0051	0.0164	0.0018	0.0123	0.715	0.479	0.932	0.228
	13	0.0004	0.0109	0.0014	0.0155	−0.235	0.815	0.932	0.075
	19	0.0006	0.0114	0.0002	0.0116	0.086	0.932	0.932	0.035
VLPFC									
	1	0.0089	0.0402	−0.0013	0.0247	0.945	0.351	0.468	0.306
	3	0.0066	0.0172	0.0024	0.0287	0.561	0.578	0.578	0.178
	18	0.0522	0.2253	−0.0024	0.0150	1.054	0.299	0.468	0.342
	20	0.0049	0.0108	0.0011	0.0121	1.038	0.306	0.468	0.331
DLPFC									
	2	−0.0048	0.0400	−0.0059	0.0281	0.097	0.923	0.961	0.032
	5	0.0137	0.0328	0.0029	0.0246	1.160	0.253	0.455	0.373
	8	−0.0004	0.0122	−0.0006	0.0192	0.050	0.961	0.961	0.012
	9	0.0281	0.0453	−0.0165	0.0368	3.364	0.002 *	0.014*	1.081
	10	0.0000	0.0152	−0.0053	0.0168	1.045	0.303	0.455	0.331
	15	0.0259	0.1052	−0.0026	0.0299	1.138	0.263	0.455	0.369
	17	0.0062	0.0293	−0.0014	0.0158	0.998	0.325	0.455	0.323
FPA									
	6	0.0036	0.0131	0.0025	0.0179	0.220	0.827	0.827	0.070
	7	0.0052	0.0095	0.0042	0.0157	0.240	0.812	0.827	0.077
	12	0.0046	0.0160	−0.0018	0.0211	1.082	0.286	0.715	0.342
	14	0.0084	0.0191	−0.0010	0.0127	1.801	0.080	0.400	0.580
	16	0.0043	0.0163	0.0005	0.0148	0.774	0.444	0.740	0.244

*p*-values were corrected for multiple comparisons using the Benjamini-Hochberg false discovery rate procedure. OFC, orbitofrontal cortex; VLPFC, ventrolateral prefrontal cortex; DLPFC, dorsolateral prefrontal cortex; FPA, frontopolar cortex; fNIRS, functional near-infrared spectroscopy; HbO, oxygenated hemoglobin; SD, standard deviation; *p < 0.05.

### Task 2: mental imagery of yoga postures

**Figure 3:**
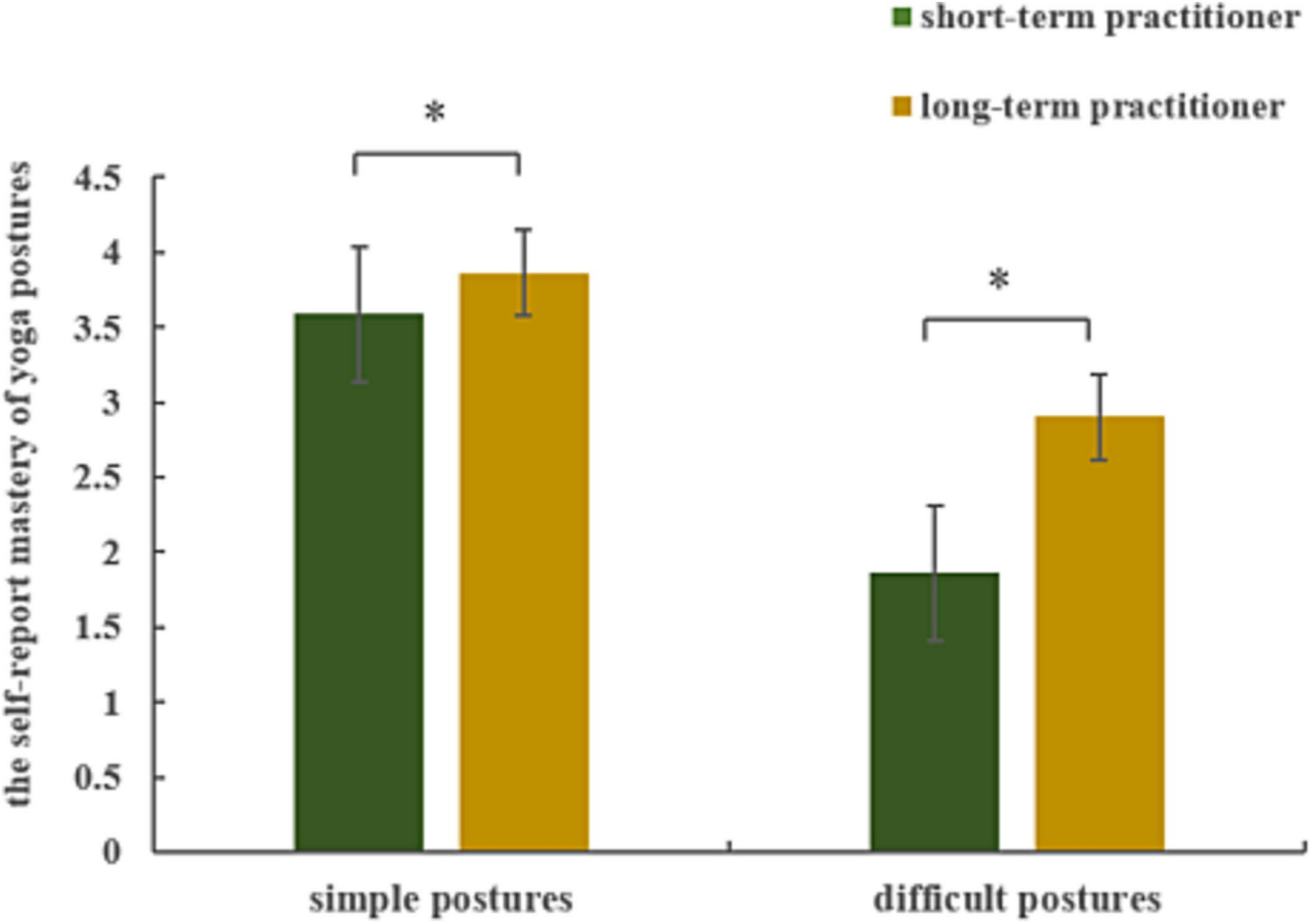
Self-reported mastery of yoga postures.

Repeated-measures ANOVA results for the self-reported mastery of yoga postures revealed a significant main effect of group (*F*_(1, 19)_ = 30.276, *p* < 0.001, $\eta_{p}^{2}$ = 0.614) and of posture difficulty level (*F*_(1, 19)_ = 468.058, *p* < 0.001, $\eta_{p}^{2}$ = 0.961), and a significant interaction between group and posture difficulty level (*F*_(1, 19)_ = 25.831, *p* < 0.001, $\eta_{p}^{2}$ = 0.576). Long-term yoga practitioners reported better mastery than short-term yoga practitioners of both simple (*p* = 0.007) and difficult (*p* < 0.001) yoga postures (see Figure 3).

**Figure 4:**
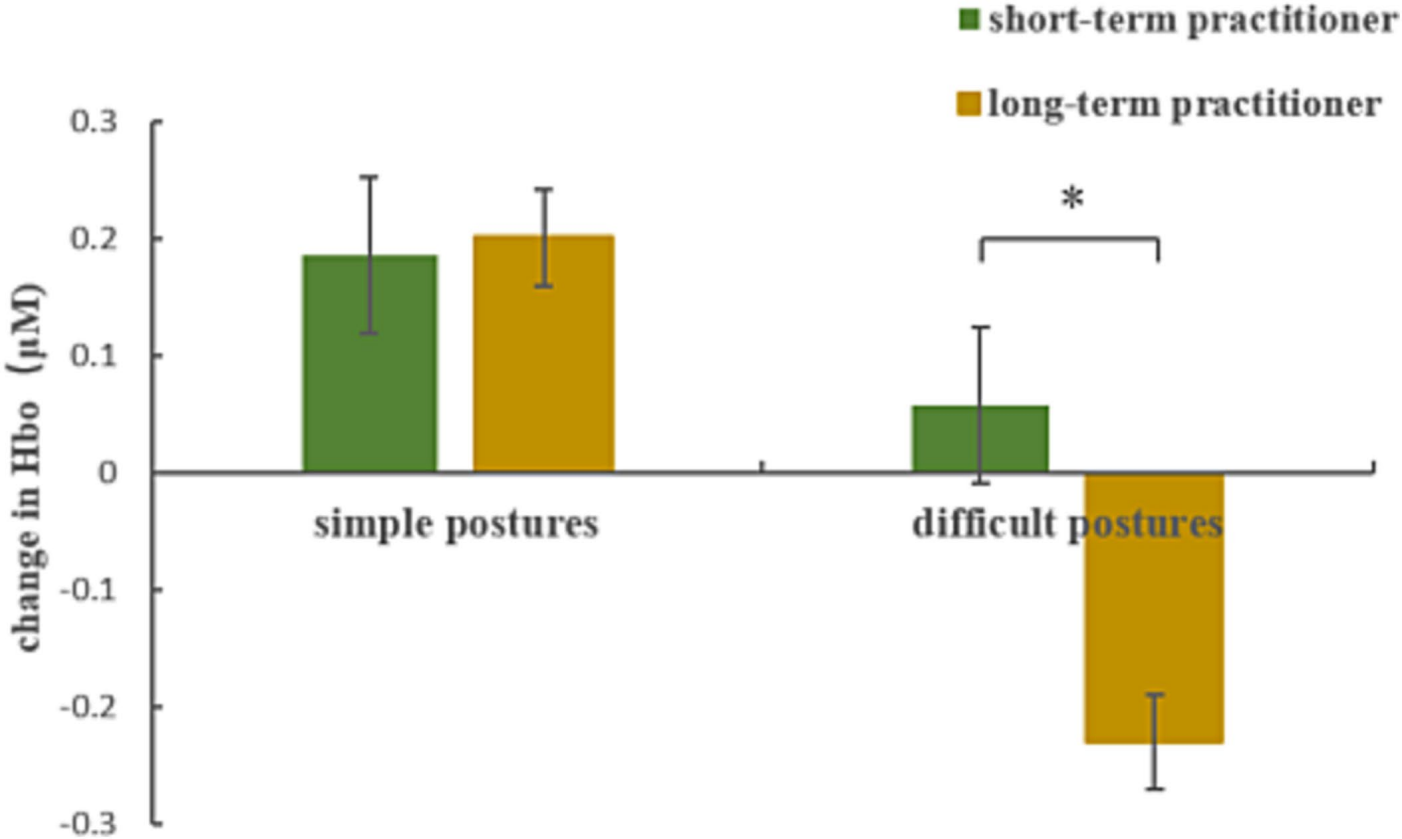
HbO concentration changes in the VLPFC (channel 3) between long-and short-term yoga practitioners during mental imagery of simple and difficult yoga postures.

Repeated-measures ANOVA results for the HbO concentration in the PFC during mental imagery of yoga postures revealed significant main effects or interactions between group and posture difficulty level in 3 (1, 3, and 20) of 20 channels. These channels were located over the VLPFC (see Table 4). After using *post hoc* tests and controlling for multiple comparisons using the Benjamini-Hochberg false discovery rate procedure, we found that the data from channel 3 remained significant (see Figure 4; Table 5) (Benjamini and Hochberg, 1995). There was a significant main effect of group (*F*_(1, 19)_ = 9.035, *p* = 0.007, $\eta_{p}^{2}$ = 0.322) and of posture difficulty level (*F*_(1, 19)_ = 9.873, *p* = 0.005, $\eta_{p}^{2}$ = 0.342). Long-term practitioners showed significantly lower activation than short-term practitioners in the VLPFC associated with the mental imagery of yoga postures. In addition, VLPFC activity elicited by mental imagery of the difficult postures was significantly lower than that elicited by the simple postures. There was also a significant interaction between group and posture difficulty level (*F*_(1, 19)_ = 5.987, *p* = 0.024, $\eta_{p}^{2}$ = 0.240). The HbO concentration in the VLPFC of long-term practitioners elicited by difficult postures was significantly lower than that of short-term practitioners (*p* = 0.008). By contrast, there was no significant difference in the VLPFC HbO concentration between these two groups during the mental imagery of simple postures (see Figure 4).

**Table 4:** Main effects and interactions during mental imagery of simple and difficult yoga postures for VLPFC activity (as assessed by HbO concentration) between long-and short-term yoga practitioners.

		Long-term practitioners		Short-term practitioners				
Area	Channel	Mean	SD	Mean	SD	Practice effect	Posture difficulty effect	Practice × posture difficulty
VLPFC								
	1							
	simple postures	−0.0432	0.0378	−0.0791	0.6531	F_(1, 19)_ = 10.283	*F*_(1, 19)_ = 0.910	*F*_(1, 19)_ = 1.208
	difficult postures	−0.0584	0.3940	0.3224	0.9905	*p* = 0.005, $\eta_{p}^{2}=$ 0.351	*p* = 0.352, $\eta_{p}^{2}=$ 0.046	*p* = 0.285, $\eta_{p}^{2}=$ 0.060
	3							
	simple postures	0.2007	0.1829	0.1857	0.3011	F_(1, 19)_ = 9.035	*F*_(1, 19)_ = 9.873	F_(1, 19)_ = 5.987
	difficult postures	−0.2301	0.3297	0.0577	0.3007	*p* = 0.007, $\eta_{p}^{2}=$ 0.322	*p* = 0.005, $\eta_{p}^{2}=$ 0.342	*p* = 0.024, $\eta_{p}^{2}=$ 0.240
	18							
	simple postures	−0.1177	0.3339	0.0330	0.3528	F_(1, 19)_ = 0.085	F_(1, 19)_ = 0.149	F_(1, 19)_ = 0.243
	difficult postures	0.0430	1.2800	−0.0206	0.5158	*p* = 0.774, $\eta_{p}^{2}=$ 0.004	*p* = 0.704, $\eta_{p}^{2}=$ 0.008	*p* = 0.628, $\eta_{p}^{2}=$ 0.013
	20							
	simple postures	0.2089	0.1948	0.1234	0.1928	F_(1, 19)_ = 1.251	F_(1, 19)_ = 15.536	F_(1, 19)_ = 7.181
	difficult postures	−0.1760	0.2414	0.0226	0.3161	*p* = 0.277, $\eta_{p}^{2}=$ 0.062	*p* = 0.001, $\eta_{p}^{2}=$ 0.450	*p* = 0.015, $\eta_{p}^{2}=$ 0.274

HbO, oxygenated hemoglobin; VLPFC, ventrolateral prefrontal cortex; SD, standard deviation; *p < 0.05.

**Table 5:** Mean changes in HbO concentrations in the VLPFC between long-and short-term yoga practitioners during mental imagery of difficult postures.

		Long-term practitioners		Short-term practitioners			
Area	Channel	Mean	SD	Mean	SD	*p*-value	Corrected-*p*
VLPFC							
	1	−0.0584	0.3940	0.3224	0.9905	0.095	0.127
	3	−0.2301	0.3297	0.0577	0.3007	0.002*	0.008*
	18	0.0430	1.2800	−0.0206	0.5158	0.860	0.860
	20	−0.1760	0.2414	0.0226	0.3161	0.026	0.052

*p*-values were corrected for multiple comparisons using the Benjamini-Hochberg false discovery rate procedure. HbO, oxygenated hemoglobin; VLPFC, ventrolateral prefrontal cortex; SD, standard deviation; *p < 0.05.

### Task 3: mindfulness meditation

The independent samples t-test results showed that the HbO concentration in the OFC of long-term yoga practitioners during mindfulness meditation was significantly higher than that of short-term yoga practitioners for channel 4 (*p* = 0.013) and channel 11 (*p* = 0.011). The HbO concentration in the VLPFC cortex of long-term yoga practitioners was significantly higher than that of short-term yoga practitioners (*p* = 0.032) (see Table 6).

**Table 6:** Mean changes in HbO concentrations between long-and short-term yoga practitioners during mindfulness meditation as assessed in 20 prefrontal fNIRS channels over four cortical areas.

		Long-term practitioners		Short-term practitioners					
Area	Channel	Mean/μm	SD	Mean/μm	SD	*t*	*p*-value	Corrected-p	Cohen’s d
OFC									
	4	0.0046	0.0130	−0.0113	0.0209	2.881	0.006*	0.013*	0.914
	11	0.0102	0.0270	−0.0129	0.0177	3.210	0.003*	0.011*	1.012
	13	−0.0025	0.0132	−0.0109	0.0197	1.579	0.123	0.164	0.501
	19	0.0013	0.0119	−0.0042	0.0146	1.326	0.193	0.193	0.413
VLPFC									
	1	0.0007	0.0106	−0.0038	0.0067	1.548	0.131	0.174	0.507
	3	0.0022	0.0133	−0.0087	0.0202	1.960	0.058	0.116	0.637
	18	0.0017	0.0194	−0.0032	0.0110	0.924	0.362	0.362	0.311
	20	0.0037	0.0126	−0.0059	0.0095	2.741	0.010*	0.032*	0.86
DLPFC									
	2	0.0011	0.0077	0.0000	0.0227	0.188	0.852	0.852	0.065
	5	0.0080	0.0134	−0.0014	0.0134	1.752	0.093	0.608	0.701
	8	−0.0027	0.0131	−0.0055	0.0134	0.667	0.509	0.852	0.211
	9	−0.0061	0.0159	0.0158	0.0504	−1.172	0.261	0.608	0.586
	10	0.0028	0.0172	0.0051	0.0318	−0.268	0.790	0.852	0.090
	15	−0.0019	0.0215	−0.0051	0.0151	0.441	0.663	0.852	0.172
	17	0.0075	0.0294	−0.0030	0.0140	1.383	0.175	0.608	0.456
FPA									
	6	−0.0024	0.0297	−0.0063	0.0111	0.539	0.593	0.593	0.174
	7	−0.0009	0.0213	−0.0052	0.0100	0.773	0.445	0.556	0.258
	12	0.0032	0.0160	−0.0029	0.0131	1.325	0.193	0.322	0.417
	14	0.0009	0.0169	−0.0062	0.0120	1.460	0.153	0.322	0.484
	16	0.0027	0.0120	−0.0032	0.0139	1.444	0.157	0.322	0.454

*p*-value was corrected for multiple comparisons using the Benjamini-Hochberg false discovery rate procedure. OFC, orbitofrontal cortex; VLPFC, ventrolateral prefrontal cortex; DLPFC, dorsolateral prefrontal cortex; FPA, frontopolar cortex; fNIRS: functional near-infrared spectroscopy; HbO, oxygenated hemoglobin; SD, standard deviation; *p < 0.05.

## Discussion

The aim of this study was to characterize neural responses to three yoga-specific components: breath work, yoga postures, and mindfulness mediation. Participants with long-and short-term yoga practice experience completed abdominal breathing, mental imagery of yoga postures, and mindfulness meditation while fNIRS data were recorded. The results supported one of our hypotheses, namely, that experienced yoga practitioners showed similar but also unique neural activities in the various subdivisions of the PFC during the three yoga tasks. Hemodynamic changes in the DLPFC improved only during abdominal breathing, and OFC activity increased only during mindfulness meditation. The activity in the VLPFC changed during both mental imagery of postures and mindfulness meditation. To the best of our knowledge, this is the first demonstration of fNIRS comparing the neural characteristics of different yoga components. Consistent with our results, Desai et al. (2015) reviewed 15 studies using electroencephalography and found that breathing, meditation, and posture practice elicited similar but unique enhancements of brain wave activity. Alpha waves improved in amplitude and frequency during all three yoga components; the amplitude and frequency of beta waves increased only during breathing, and theta wave activity improved after both posture practice and breathing (Desai et al., 2015). Our hypothesis that the three components of yoga training would enhance activity in the corresponding PFC area was not fully accurate. During abdominal breathing and mindfulness meditation, we observed higher PFC activity in long-term yoga practitioners than in short-term yoga practitioners. However, long-term yoga practitioners showed much lower activity in the VLPFC compared with short-term yoga practitioners. Below, we discuss this finding further.

### Breath work

Our fNIRS data showed that the HbO concentration in the DLPFC of long-term yoga practitioners was significantly higher than that of short-term yoga practitioners during abdominal breathing. The increased HbO concentration may be due to better slow breath control that led to better perfusion and oxygenation in long-term yoga practitioners. This may be a mechanistic underpinning of the deep abdominal breathing control benefits from long-term yoga experience. An enhanced ability to control breathing has been related to physical and mental health in daily life (Stutz and Schreiber, 2017). Zaccaro et al. (2022) compared the aftereffects of slow nasal breathing with a session of mouth breathing at the same respiratory rate. They showed that slow breathing modulates brain activity and hence the subjective experience to the point of inducing a non-ordinary state of consciousness (Zaccaro et al., 2022). The increased HbO concentration in the DLPFC may also represent a benefit of long-term yogic breathing experience on DLPFC function. DLPFC is a cognition area responsible for planning, organizing, and regulating and is closely related to functions such as attention, memory, and emotional control (Hertrich et al., 2021; Wischnewski et al., 2021). Studies have provided strong evidence for the advantages of regular yogic breathing on cognition. Yogic breathing has shown benefits for verbal and spatial cognition, memory, sustained attention and emotional regulation (Marshall et al., 2014; Ma et al., 2017). The current study provides new neural evidence for the cognitive benefits of yogic breathing.

### Mental imagery of yoga postures

The self-assessment scores of yoga posture mastery indicated that the mastery levels of long-term yoga practitioners on both simple and difficult postures were higher than those of short-term yoga practitioners. Consistent with the present result, a previous study also observed a posture control advantage suggesting possible benefits in supraspinal feed-forward motor adaptations associated with yoga training (Pinto et al., 2022).

Inconsistent with our hypotheses, the present fNIRS data showed that the activation level of the VLPFC was significantly lower in the long-term yoga practitioners than in the short-term yoga practitioners during mental imagery of difficult yoga postures. The postures we selected for the mental imagery task may have impacted the neural results. Dybvik and Steinert (2021) used fNIRS to explore brain activity when participants actually practiced yoga postures and found that brain activation was significantly higher in difficult postures compared with simple postures. The inconsistency across studies for these results may be due to the different neural underpinnings associated with the two experimental paradigms. It is likely that using imagery for performing the postures in our study versus actually performing the postures as in the study by Dybvik et al. involve different neural correlates. Our findings suggest that long-term experienced practitioners required less neural activity to image more difficult postures than short-term yoga practitioners, which may have largely benefited from long-term posture training experience. Another previous study found that long-term exercise facilitated neuroplasticity associated with brain functions (Hötting and Röder, 2013). The VLPFC is part of a default mode network involved in self-awareness. Evidence indicates that activation of the default mode network is stronger during a resting state and is significantly decreased during target tasks (Sheline et al., 2009). Consistent with these observations, the lower activation in the VLPFC observed during mental imagery of yoga postures in the present study may indicate more efficient neural recruitment. Findings in a study by Hertrich et al. (2021) suggested that the cognitive load associated with difficult postures is greater than that for simple postures during the actual performance. The more difficult the posture, the greater the cognitive load and the stronger the corresponding PFC activation. Cecchini et al. (2016) found that motor imagery is developed linked to the development of motor skills. Therefore, enhanced activation in the PFC during actual performance of postures may be a reasonable interpretation for efficient neural recruitment during the mental imagery of postures.

### Mindfulness meditation

The HbO concentration in the OFC and VLPFC of long-term yoga practitioners during mindfulness mediation was significantly higher than that of short-term yoga practitioners. The results confirmed our hypotheses that yogic meditation training experience would amplify activities in the corresponding PFC area. Consistent with our results, Nascimento et al. (2018) reported increased activation of the PFC, especially in the OFC and VLPFC, during meditation. Other studies have also found a close neural association between meditation and these two brain areas. Kong et al. (2016) found that mindfulness was positively associated with OFC activation. Kurth et al. (2023) observed a negative relationship between age and OFC, and surprisingly, age-related declines in the OFC is diminished in meditation practitioners. Mooneyham et al. (2016) reported that mindfulness meditation was associated with the VLPFC. Meditation practice also enhanced the functional connectivity of the VLPFC to other brain regions (Barrós-Loscertales et al., 2021). Taken together, these studies indicate the neural benefits associated with yogic mindfulness meditation practice experience. Our findings provide additional neural evidence for the many behavioral studies showing the advantages of mindfulness meditation. Numerous studies have found that mindfulness meditation benefits mental refreshment, attention, emotional control, and self-awareness, which are associated with the OFC and VLPFC (Shen et al., 2020; Miyashiro et al., 2021; Shen et al., 2023).

## Limitations

The present study has some limitations. First, the present study lacks scientific behavioral assessments. Thus, it was not possible to connect the neural advantages of each yoga component with the corresponding behavioral advantage, reducing the significance of this study for practical application. Future research exploring the benefits of yoga should combine accurate behavioral measurements for breathing, posture imagery, and mindfulness meditation with neural indicators. Second, although we balanced the two groups for age and educational level, differences between the two groups beyond yoga training may still have confounded the results. Future research should recruit participants with no yoga experience and conduct long-term yoga interventions to more accurately explore the neurobehavioral benefits of yoga. Third, each of the three yoga components can be further subdivided into several categories, the benefits of which should be further explored in future research.

## Conclusion

PFC activation, as assessed using HbO concentrations during fNIRS, showed some similarities as well as differences during the performance of the three core components of yoga practice, namely, yogic breathing, posture imagery, and mindfulness meditation. Long-term yoga practice experience was associated with the neural benefit of efficient activation in the PFC. Long-term mindfulness mediation experience improved brain activity in both the OFC and VLPFC, whereas long-term yogic breathing improved brain activity in the DLPFC. Long-term yoga posture practice experience was associated with efficient neural recruitment in the VLPFC, as reflected by lower activation during mental imagery of yoga postures.

## Data availability statement

The original contributions presented in the study are included in the article/supplementary material, further inquiries can be directed to the corresponding author.

## Ethics statement

The studies involving humans were approved by the Shanghai University of Sport Ethics Committee and was performed in accordance with the ethical standards laid down in the 1964 Declaration of Helsinki and its later amendments. The studies were conducted in accordance with the local legislation and institutional requirements. A total of 40 women were recruited from yoga studios in shanghai China. Written informed consent for participation was not required from the participants or the participants’ legal guardians/next of kin in accordance with the national legislation and institutional requirements.

## Author contributions

XL: Conceptualization, Funding acquisition, Methodology, Writing – original draft, Investigation, Supervision. YZ: Formal analysis, Investigation, Writing – review & editing. CZ: Conceptualization, Investigation, Methodology, Writing – review & editing. HW: Methodology, Resources, Writing – review & editing. XW: Conceptualization, Funding acquisition, Methodology, Supervision, Validation, Writing – review & editing.
